# Population Structure and Dispersal Patterns within and between Atlantic and Mediterranean Populations of a Large-Range Pelagic Seabird

**DOI:** 10.1371/journal.pone.0070711

**Published:** 2013-08-12

**Authors:** Meritxell Genovart, Jean-Claude Thibault, José Manuel Igual, Maria del Mar Bauzà-Ribot, Corinne Rabouam, Vincent Bretagnolle

**Affiliations:** 1 Population Ecology Group, Institut Mediterrani d'Estudis Avançats IMEDEA (CSIC-UIB), Esporles, Mallorca, Spain; 2 Muséum National d'Histoire Naturelle, Département Systématique et Evolution, UMR7205 Origine, Structure et Evolution de la Biodiversité, Paris, France; 3 Centre d'Etudes Biologiques de Chizé, CNRS UPR 1934, Villiers en Bois, France; University of Plymouth, United Kingdom

## Abstract

Dispersal is critically linked to the demographic and evolutionary trajectories of populations, but in most seabird species it may be difficult to estimate. Using molecular tools, we explored population structure and the spatial dispersal pattern of a highly pelagic but philopatric seabird, the Cory's shearwater *Calonectris diomedea*. Microsatellite fragments were analysed from samples collected across almost the entire breeding range of the species. To help disentangle the taxonomic status of the two subspecies described, the Atlantic form *C. d. borealis* and the Mediterranean form *C. d. diomedea*, we analysed genetic divergence between subspecies and quantified both historical and recent migration rates between the Mediterranean and Atlantic basins. We also searched for evidence of isolation by distance (IBD) and addressed spatial patterns of gene flow. We found a low genetic structure in the Mediterranean basin. Conversely, strong genetic differentiation appeared in the Atlantic basin. Even if the species was mostly philopatric (97%), results suggest recent dispersal between basins, especially from the Atlantic to the Mediterranean (aprox. 10% of migrants/generation across the last two generations). Long-term gene flow analyses also suggested an historical exchange between basins (about 70 breeders/generation). Spatial analysis of genetic variation indicates that distance is not the main factor in shaping genetic structure in this species. Given our results we recommend gathering more data before concluded whether these taxa should be treated as two species or subspecies.

## Introduction

Dispersal is a central concept in population and evolutionary biology [1]. Two types of animal dispersal are commonly recognised: “natal dispersal”, i.e. the movement between the natal area and the area where breeding first takes places, and “breeding dispersal”, i.e. the movement between successive breeding areas. In both cases, dispersal may result in gene flow, defined as the movement and integration of genes from one population to another [2]. Both dispersal and gene flow are closely linked to the demographic and evolutionary trajectories of populations, [3] and their accurate quantification is essential for basic as well as applied sciences. Additionally, dispersal, and thus gene flow, may change over space and time [1] and therefore the distinction between historical and present dispersal processes is crucial to evaluate its importance at evolutionary and ecological time scales. However, dispersal is often very difficult to estimate by capture-recapture methods, especially in seabirds, which often breed in large colonies on remote islands or cliffs [4], and furthermore direct measures of dispersal may not necessarily reflect gene flow. Molecular tools may provide an alternative method for assessing effective dispersal patterns in seabird species [5]–[8]. Highly variable DNA markers such as microsatellites allow measuring genetic differentiation within and among populations but also detailed and direct estimates of gene flow, and consequently historical, as well as current migration patterns can be inferred [9].

The Mediterranean Sea became separated from the Atlantic Ocean during the Messinian salinity crisis, approximately 5.5My ago and the present day species inhabiting the Mediterranean are mostly the result of subsequent colonization, mainly from the Atlantic Ocean [10]. A recent review analysed patterns of genetic isolation between these two basins for several marine species [11]. Species in the Mediterranean Sea did not show a uniform phylogeographical pattern, finding any combination of two extreme cases: from complete genetic separation between Atlantic–Mediterranean populations since the early Pliocene to complete absence of population differentiation, usually following late Pleistocene recolonization. Unfortunately marine birds were not included in that study. Actually very few investigations were conducted on genetic variation in seabirds between Atlantic and Mediterranean populations, and with the exception of one study on yellow-legged gull *Larus michahellis* [12] that also included microsatellite analysis, they were all based on mtDNA ([13] working on storm-petrel *Hydrobates pelagicus*, and [14] on Cory's shearwater).

In this study we investigate patterns of genetic variation at nuclear loci in a large-range pelagic seabird, the Cory's shearwater *Calonectris diomedea* throughout its breeding range. In particular we investigate differences between the two described taxa, the larger one breeding in the Atlantic (mean mass 790 g.) and the smaller one, breeding on the Mediterranean islands (mean mass 650) [15]. Previous genetic studies on this species complex used blood proteins [16], DNA fingerprinting [17], [18] and mtDNA [14], [19]. The demography of Cory's shearwater has been extensively studied at local level through ringing history, without considering dispersal processes between colonies and populations (see [20] for a review, [21]–[23]) but occasionally reporting observations of birds ringed at other colonies. The results of previous genetic and demographic studies are contradictory: i) both ringing studies and genetic analyses revealed a strong philopatric behaviour in this species, with short-distance dispersal occurring mainly between sub-colonies within local populations [24]–[26]; ii) using mtDNA, Mediterranean and Atlantic populations are genetically distinct, with long-time geographic isolation and gene flow barriers since the mid Pleistocene [14], [19], but iii) ringing studies over the last 25 years revealed numerous exchanges of individuals (either immatures and adults) between Mediterranean and Atlantic populations, some of them with documented successful breeding in the new colony [14], [27]–[31]. However, the demographic importance of these exchanges is difficult to evaluate due to the difficulty in detecting dispersal events because only a very small proportion of birds are ringed and in a limited number of colonies.

The aim of this study is to explore population structure and the spatial dispersal pattern in the Cory's shearwater and to infer short and long term dispersal between the two ocean basins. For that purpose, we genetically analyse the largest and most comprehensive data set so far used in this species, i.e. 387 individuals sampled from 27 breeding colonies from most Mediterranean and some Macaronesian colonies.

## Materials and Methods

### Ethics Statement

All animals were handled in strict accordance with good animal practice as defined by the current European legislation, and all animal work was approved by the respective national and regional committees for scientific capture (Organismo Autónomo de Parques Nacionales (Spain), Ministerio de Medio Ambiente y Medio Rural (Spain), Govern Balear (Spain), Centre de Recherches par le Baguage des Populations d'Oiseaux, (France), Department of Environment (Greece) and Istituto Nazionale per la Fauna Selvatica (Italy)).

### Study species and sampling

Cory's shearwater is a pelagic seabird that breeds mainly on islands, throughout the Mediterranean Sea as well as the Atlantic Islands of Berlenga, Selvagens, Canaries, and Azores. New colonies have also been discovered recently along the coasts of Galicia [32] and Aquitaine, France [33]. Currently the taxonomy of Cory's shearwater is unclear. Cape Verde shearwater *C. edwarsii* (not considered here) appears to be distinct and is widely treated as a separate species [34]. The Mediterranean subspecies *Calonectris diomedea diomedea* shows some morphological, ecological, vocal and genetic differences from the north Atlantic subspecies *C. d. borealis* [14], [19], [20], [35], prompting some authors to treat them as separate species [36]. Nevertheless the degree of overlap between diomedea and borealis and evidence of inter-colony movement from a small number of individuals indicates that the relationship between these taxa is unclear.

**Figure 1:**
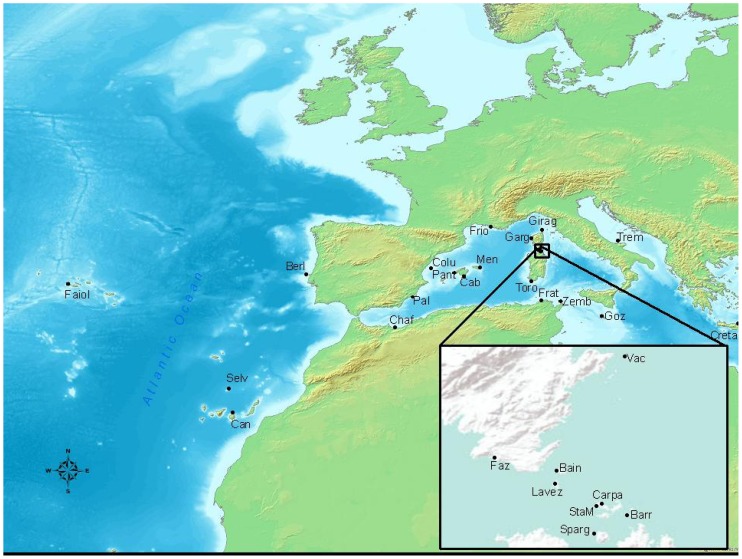
Location of sampled colonies of Cory's shearwater *Calonectris diomedea*. Colony abbreviations are, i) for Mediterranean colonies: Creta (Cre), Tremiti (Trem), Gozzo (Goz), Zembra (Zem), Galitte (Gal), Toro (Tor), Sparggiotto (Spa), Barretini (Barr), Carpa (Car), Santa Maria (StaM), Fazzio (Faz), Lavezzi (Lav), Gargallo (Gar), San Bainso (Bain), Vacca (Vac), Giraglia (Gir), Frioul (Fri), Menorca (Men), Cabrera-Na Foradada (For), Pantaleu (Pant), Columbretes (Col), Palomas (Pal), Chafarinas (Chaf), ii) and for Atlantic colonies: Berlenga (Berl), Canarias (Can), Selvagem (Selv) and Azores- Faiol (Fai).

We visited 27 Cory's Shearwater breeding colonies throughout almost all of its breeding range in the Mediterranean and Atlantic regions (Figure 1) and took blood samples from 387 birds captured in colonies during the breeding period; most sampled birds were breeding adults but some chicks were also sampled. We did not sample chicks and adults from the same colony to avoid sampling related birds. A small blood sample (ca. 50 µL) was taken from the femur vein of the bird, collected in a capillary tube and transferred to a tube with ethanol. All birds were released at the same place they were caught, no animals were sacrificed and no negative effects have ever been observed with this sampling protocol.

### DNA extraction and amplification

Total DNA was isolated from blood samples by overnight incubation at 55°C in SET buffer with 30 µl SDS 10% and 2.5 units/ml of proteinase K followed by a standard phenol/chloroform protocol [37]. DNA was resuspended in TE buffer [41]. Microsatellite loci previously designed for the Balearic shearwater *Puffinus mauretanicus* [38] were used for genetic analysis. We were able to amplify nine microsatellites, of which six were polymorphic (see Table S1). Amplification reactions were performed in a total volume of 10 µl with 0.4 µM of each primer (fluorescence labelled with VIC, NED6, FAM, PET and FAM), 0.2 mM dNTP, 1x Taq buffer, 1 U of Taq DNA polymerase (Bioline), 2–3 mM of MgCl_2_ (depending on the primer pair) and 1–2 µl of template DNA. The thermocycling conditions were as follows: 94°C for 2 min, followed by 34 cycles of 95°C for 30 seconds, 50°C–60°C for 30 seconds and 72°C for 30 seconds, with a final extension of 72°C for 5 min. Specific annealing temperatures and magnesium concentrations for each locus are shown in Table S1. We checked the amplification and purification results by loading 1–2 µl of product in a 1.5% agarose gel. Reactions were loaded together and the length of the DNA fragments were analysed directly from PCR product using an ABI 3100 automated sequencer (Applied Biosystems, Warrington, UK) and the ABI software GeneMapper v. 3.7 and visually rechecked. Alleles were scored as PCR product size.

### Genetic variability

For 24 breeding colonies (sample sizes ≥9), we measured the mean number of alleles per locus, and the intrapopulation genetic diversity for each population was evaluated in terms of allelic richness as well as observed and unbiased expected heterozygosity [39] using the software Genetix v. 4.05 [40] and Fstat v. 2.9.3.2 [41]. With the software Genetix v. 4.05, we also calculated the inbreeding coefficient (F_IS_) and tested for deviations from the Hardy-Weinberg equilibrium. Using permutations (>1000) we tested for the occurrence of non-random associations of pairs of loci (i.e. linkage disequilibrium). A deficit in heterozygotes can be mimicked by null alleles; this was checked by assuming that some of the homozygotes were heterozygotes for the null allele and that individuals failing to amplify were homozygous for the null allele. Additionally with the software FreeNA [42] we computed a global F_ST_ using the ENA correction method and using the original data. The ENA correction method was found to efficiently correct for the positive bias induced by the presence of null alleles on F_ST_ estimation and provide accurate estimation of F_ST_ in presence of null alleles [42].

### Gene flow and genetic structure

To asses genetic differentiation we used F-statistics [43]–[45] and the test of differentiation implemented in Genepop [46]. We derived an F_ST_ pairwise distance matrix between sampling localities [47] and estimated their significance levels using permutation tests (>1000 times) with Arlequin v.3.1 [48]. To reduce the probability of Type I errors, we used Benjamini-Yekutieli corrections in tests involving multiple comparisons [49].

Population structure was further analysed using the Bayesian assignment method implemented in Structure v.2.3 [50]. This program assumes a model with a specific number of populations (*K*) and estimates the probability of the data (*X*) being associated to this specific number of populations (the log likelihood value *Pr* (*X|K*)). Estimation of K was based on Evanno's method [51]. However, we also used the log probability of the data given to discard K = 1, a possibility that cannot be a priori ruled out in our case, and which cannot be measured with Evanno's method. The admixture ancestry model was run with the assumption of correlated allele frequencies to improve the clustering of closely related populations [52]; we used the most recent version of this program that allows weak population structure to be inferred with the assistance of sampling information [53], considering each colony as a different sampling location. To estimate the number of subpopulations (K), ten independent runs, for each value between K = 1 to K = 15 were carried out at 1*10^6^ Markov Chain Monte Carlo (MCMC) repetitions and a burn-in period of 100,000 iterations. For visualising and compare different Structure results for different K values we used the software the Software Distruct 1.1 [54].

To reveal if there was a genetic structure within the data set we also conducted an analysis of molecular variance (AMOVA, [55]) with the program Arlequin v.3.1 [48] and to test for different partitioning of genetic variation in the species, a hierarchical analysis of molecular variance (AMOVA, [55]) based on the number of different alleles was performed with Arlequin v.3.1 [48]. Statistical significance was determined by >1000 permutations of the genotypes. Taking into account previous results on genetic differentiation and population structure, we conducted four AMOVA analysis using different types of hierarchical groupings: a) two groups corresponding to the Atlantic and the Mediterranean colonies, b) three groups: one corresponding to the Mediterranean colonies and the other two to the Atlantic colonies (the Azores and Selvagem in one group, and Berlenga and the Canaries in the other) c) three groups: one corresponding to the Mediterranean colonies other than those from Spargiotto and Barretini, another corresponding to Azores and Selvagem, and another corresponding to Berlenga, Canaries, Spargiotto and Barretini; and d) four groups: the Mediterranean colonies subdivided in two groups, with birds from Barretini and Spargiotto separated from the others, and the two Atlantic groups as previously described.

To avoid overparametrization (i.e. to include more parameters in the model than can be estimated from the data), we pooled all Mediterranean samples and all the Atlantic samples to obtain an estimate of the magnitude and direction of dispersal rates between the Atlantic and the Mediterranean. We used two methods: first, we estimated the migration rate (m) using the Bayesian assignment algorithm implemented in BayesAss [56] to specifically consider short-term gene flow (i.e. during the past one-to-three generations). While Structure uses a Bayesian probabilistic model to assign individuals to clusters, BayesAss estimates the posterior probability of an individual's migratory history and thus allows estimating the rate and direction of recent dispersal. Unlike estimators of long-term gene flow, BayesAss makes relatively few assumptions about demography and can be applied to populations that are not in the Hardy-Weinberg equilibrium. The MCMC method was run for 20,000,000 iterations with a burn-in period of 1,000,000 and a sampling frequency of 2,000 iterations. Delta values (i.e. maximum parameter change per iteration) were adjusted on the basis of preliminary runs (delta values ranging from 0.05 to 0.30) to optimize the terminal proposed changes between chains (40% to 60% of the total iterations) and to ensure that sufficient parameter space was searched [57]. Our final run used delta values of 0.05, 0.10, and 0.30 for allele frequency, migration and inbreeding respectively. Secondly, we used the Bayesian coalescent approach implemented in Migrate 3.2 [58]–[59] to estimate the mutation-scaled population size theta, (theta  = 4N_e_μ, where N_e_ denotes the effective population size and μ the mutation rate per locus per generation), and the mutation- scaled immigration rate (M) (M =  m/μ, where m denotes immigration rate size and μ the mutation rate per locus per generation). We ran Migrate using a Brownian motion mutation model with constant mutation rates and starting parameters based on F_ST_ calculations An uniform prior distribution (min = 0, max = 500, mean  = 250) was used to estimate theta, and a uniform prior distribution (min = 0, max = 1000, mean  = 500, delta  = 100) was used for M. The priors were chosen based on the performance of multiple trial runs with different prior values. Runs visited a total of 2,000,000 parameter values including a 500,000 burn-in period, and sampled the parameter value every 20 iterations. To assist with convergence, we used the ‘static heating scheme’ option with four concurrent chains. We evaluated convergence by looking at the effective sample size, the autocorrelation, and the posterior distribution histograms over all loci. We report the median values of theta and M, and the 0.025 and 0.975 posterior distribution values as 95% confidence interval estimates [57] of the median. As theta and M estimates from Migrate are compounded by the mutation rate, to avoid making an assumption about mutation rates, the effective number of immigrants per generation in each basin was calculated by multiplying M by theta.

To avoid estimation problems caused by very different sample sizes between groups, for both analyses BayesAss and Migrate, we randomly reduced the larger Mediterranean data set to 100 individuals.

Patterns of differentiation among populations were visualized by factorial correspondence analysis of multilocus scores (MCA) computed using Genetix v. 4.05 (6 loci, 2 factors) [40]. Conventionally, the first axis is the one that contributes most into total inertia, and usually reveals the differentiation between species and subspecies [60]. Given the large number of birds involved in the analysis, the MCA output was simplified by plotting the centre of gravity of the groups of individuals defined at the spatial scale of choice, as proposed by the software Genetix [40].

### Spatial analysis of genetic data

We used the previously estimated Slatkin's F_ST_ (θ) matrix of genetic distances among colonies to calculate a new matrix θ/(1−θ) of genetic distances. A matrix of geographical distances was also calculated as the natural logarithm of the shortest distance from colonies over the sea (shearwaters do not fly over land). To search for evidence of isolation by distance, we first compared the genetic and geographical distance matrices using Mantel tests with software R and the ape package [61]. The Mantel test is a permutational procedure used to test the statistical significance of matrix correlations [62], [63] and is widely used in population genetics because high correlations between genetic and geographical distance matrices in continuous space are thought to result from isolation by distance (IBD) processes [64]–[67]. We also used Genepop to compute the regression line describing the relationship between both distance matrices [46], [68]. Additionally, we plotted θ/(1−θ) versus the natural logarithm of the shortest distance from colonies over the sea.

Spatial analyses were carried out at two different scales: a) in the global range, including both Atlantic and Mediterranean colonies and b) at the Mediterranean scale, only including those individuals from Mediterranean colonies.

## Results

### Genetic variability

We included 387 Cory's shearwaters from 4 Atlantic and 20 Mediterranean colonies in the genetic analysis (Table 1). The six polymorphic microsatellite loci had an overall mean heterozygosity across all samples and loci of 0.50±0.24 (Table 1) and ranged from 0.35 in Berlenga to 0.59 in Fazzio (Corsica). The mean number of alleles per locus was 4.85±1.48, and the mean allelic richness was 2.86 (Table 1). The loci are inherited independently as no significant linkage disequilibrium was detected. The loci showing the highest proportions of heterozygote deficiency did not show higher proportions of non-amplifications thus not indicating the presence of null alleles (Table 1). We found F_IS_ values significantly greater than zero in most populations (Table 1).

**Table 1:** Sample sizes and genetic diversity descriptors, globally and at population level in colonies of Cory's shearwater.

Colonies	N	P	a	*A*	Ho (SD)	He (SD)	F_IS_ (IC95%)
**Mediterranean**							
Creta (Grece)	20	6/6	4.2	2.74	0.39+/−0.23	0.51+/−0.25	0.23 (0.06–0.34)
Tremiti (Adriatic)	15	6/6	4	2.69	0.27+/−0.19	0.47+/−0.25	0.43 (0.22–0.57)
Gozo (Malta)	20	6/6	4.5	2.83	0.37+/−0.27	0.48+/−0.28	0.23 (0.02–0.37)
Zembra (Tunisia)	6						
Galitte (Tunisia)	10	5/5	4	2.55	0.34+/−0.22	0.55+/−0.25	0.39 (0.1–0.51)
Toro (Sardinia)	6						
Spargiotto (Sardinia)	17	6/6	4.2	2.80	0.43+/−0.35	0.50+/−0.29	0.14 (−0.05–0.25)
Barrettini (Sardinia)	17	6/6	4.3	2.58	0.41+/−0.29	0.42+/−0.29	0.02 (−0.15–0.12)
Carpa (Sardinia)	20	6/6	4	2.91	0.46+/−0.24	0.56+/−0.26	0.19 (0.001–0.32)
Sta. Maria (Sardinia)	15	6/6	3.5	2.63	0.36+/−0.22	0.49+/−0.24	0.27 (0.03–0.45)
Fazzio (Corsica)	10	4/3	4	2.78	0.37+/−0.21	0.59+/−0.18	0.38 (−0.01–0.60)
Lavezzi (Corsica)	15	6/6	4	2.60	0.27+/−0.25	0.41+/−0.32	0.36 (0.13–0.53)
Gargalo (Corsica)	10	5/5	3.7	2.59	0.36+/−0.35	0.40+/−0.31	0.10 (−0.20–0.25)
San Bainso (Corsica)	15	6/6	4.2	2.79	0.44+/0.27	0.49+/−0.27	0.16 (−0.16–0.26)
Vacca (Corsica)	15	5/5	3.8	2.74	0.37+/−0.26	0.48+/−0.29	0.24 (0.03–0.36)
Giraglia (Corsica)	13	3/3	3.5	2.58	0.40+/−0.24	0.47+/−0.24	0.16 (−0.11–0.34)
Frioul (Marseille)	5						
Maó (Menorca)	25	4/4	4.7	2.79	0.32+/−0.25	0.48+/ 0.29	0.56 (0.35–0.71)
Na Foradada (Cabrera)	15	5/5	4	2.87	0.28+/−0.22	0.54+/ 0.20	0.50 (0.21–0.68)
Pantaleu (Mallorca)	15	6/3	3.8	2.60	0.35+/−0.25	0.46+/ 0.21	0.25 (0.04–0.32)
Columbretes (Valencia)	13	4/0	4	2.33	0.45+/−0.35	0.57+/ 0.280	0.24 (−0.05–0.40)
Palomas (Murcia)	21	6/6	3.7	2.20	0.26+/−0.21	0.38+/ 0.19	0.33 (0.07–0.52)
Chafarinas (Alborán)	23	5/5	4.5	2.85	0.32+/−0.25	0.50+/−0.25	0.37 (0.21–0.46)
**Atlantic**							
Berlenga	15	6/6	3.6	2.37	0.18+/−0.20	0.35+/ 0.29	0.50 (0.23–0.69)
Canarias	20	6/6	4.3	2.55	0.31+/−0.30	0.41+/ 0.28	0.26 (0.09–0.37)
Selvagem	15	5/3	4.8	2.53	0.33+/−0.21	0.45+/−0.29	0.28 (0.001–0.47)
Faiol (Azores)	9	4/3	3.5	2.27	0.24+/−0.30	0.53+/−0.28	0.56 (0.22–0.72)
	387						

N =  individuals sampled, P: number of usable loci (less than 10% missing data)/ number of polymorphic loci, a: average number of alleles per locus, *A*  = allele richness, H_o_ and H_e_  =  observed and unbiased expected heterozygosity (Nei, 1978), and mean estimates of F_IS_ [47], followed by a 95% confidence interval (95% CI).

### Genetic structure

Global F_ST_ estimates were very similar when using the ENA correction method (0.091) as when using the original data (0.086). F_ST_ pairwise genetic differentiation between Mediterranean colonies mostly showed small genetic differences between colonies (min F_ST_: 0.00, Max. F_ST_: 0.29, mean F_ST_: 0.065; Table 2). Individuals from two Mediterranean colonies in Sardinia (Spargiotto and Barretini) were not mutually significantly different (F_ST_ = 0.02) but unexpectedly very different from all other Mediterranean colonies (mean F_ST_: 0.16). Another striking feature came from F_ST_ pairwise genetic differentiation between the four Atlantic colonies, which could be divided into two clearly different groups: there were no differences between individuals from Canaries and Berlenga (F_ST_ = 0.02) but these birds were statistically significant from those from Azores and Selvagem (mean F_ST_ = 0.20; Table 2). The test of differentiation gave us similar results (Table 2), but being more powerful to detect genetic differences between some Mediterranean colonies.

**Table 2:** Pairwise measures of genetic differentiation among twenty-four shearwaters populations.

	Cre	Trem	Goz	Gal	Spa	Bar	Car	StaM	Faz	Lav	Gar	Bain	Vac	Gir	Mao	For	Pant	Col	Pal	Chaf	Berl	Can	Selv	Fai
**Mediterranean**																								
Creta		0.42	0.08	0.15	**0.01**	**0.00**	**0.04**	**0.04**	0.14	**0.00**	0.77	1.00	**0.03**	0.98	**0.03**	0.06	1.00	**0.01**	**0.00**	**0.00**	**0.00**	**0.00**	**0.00**	**0.00**
Tremiti	0.03		0.69	0.07	**0.01**	0.15	**0.02**	0.12	1.00	1.00	1.00	1.00	0.56	**0.04**	1.00	1.00	1.00	0.42	**0.00**	**0.00**	0.27	**0.00**	0.16	0.17
Gozo	**0.07**	0.04		0.13	**0.00**	**0.00**	0.05	1.00	1.00	1.00	1.00	1.00	0.09	0.73	1.00	0.31	1.00	1.00	**0.01**	**0.00**	**0.00**	**0.00**	**0.00**	**0.00**
Galitte	0.07	**0.11**	0.07		**0.00**	**0.00**	0.02	0.34	1.00	0.52	1.00	1.00	1.00	0.73	1.00	0.23	1.00	1.00	**0.01**	**0.00**	**0.00**	**0.00**	0.10	0.13
Spargiotto	**0.06**	**0.1**	**0.17**	**0.19**		1.00	0.06	**0.00**	**0.00**	**0.00**	**0.00**	**0.00**	**0.00**	**0.00**	**0.00**	**0.00**	**0.00**	**0.00**	**0.00**	1.00	**0.00**	**0.00**	**0.00**	**0.00**
Barrettini	**0.08**	**0.09**	**0.19**	**0.26**	0.02		**0.02**	**0.00**	**0.00**	**0.00**	**0.00**	**0.00**	**0.00**	**0.00**	**0.00**	**0.00**	**0.00**	**0.00**	**0.00**	1.00	**0.02**	**0.00**	**0.00**	**0.00**
Carpa	0.02	0.05	**0.05**	0.07	**0.07**	**0.1**		**0.04**	0.41	**0.04**	0.06	1.00	**0.02**	**0.01**	**0.03**	**0.01**	0.24	**0.00**	**0.00**	**0.00**	**0.00**	**0.00**	**0.00**	**0.00**
Sta. Maria	0.06	0.04	0.02	0.04	**0.12**	**0.17**	0.04		1.00	1.00	0.49	1.00	0.14	0.09	1.00	0.33	1.00	0.92	**0.03**	**0.00**	**0.00**	**0.00**	**0.00**	**0.00**
Fazzio	0.03	0.01	0.02	0.02	**0.14**	**0.16**	0.02	0.01		1.00	1.00	1.00	1.00	0.16	1.00	1.00	1.00	1.00	**0.00**	**0.00**	**0.01**	**0.00**	**0.01**	0.09
Lavezzi	**0.1**	0.05	0.01	0.08	**0.23**	**0.25**	0.08	0.03	0.01		0.64	1.00	0.57	0.92	1.00	1.00	1.00	1.00	**0.03**	**0.00**	**0.00**	**0.00**	0.40	0.17
Gargalo	0.05	0.06	0.02	0.02	**0.22**	**0.25**	0.07	0.06	0.03	0.02		1.00	1.00	1.00	1.00	0.74	1.00	1.00	**0.01**	**0.00**	0.05	0.05	1.00	1.00
San Bainso	0.02	0.01	0.00	0.03	**0.12**	**0.14**	0.02	0.01	−0.02	0.02	0.01		1.00	0.58	1.00	0.93	1.00	1.00	0.90	**0.00**	**0.00**	**0.00**	**0.00**	**0.01**
Vacca	0.05	0.06	0.04	−0.03	**0.15**	**0.21**	0.04	0.03	0.00	0.06	0.02	0.01		0.09	1.00	0.15	0.98	0.34	**0.00**	**0.00**	**0.00**	**0.00**	**0.01**	**0.01**
Giraglia	0.05	**0.09**	0.04	0.02	**0.21**	**0.26**	0.05	0.05	0.02	0.04	−0.01	0.03	0.03		0.84	0.95	1.00	0.34	**0.00**	**0.00**	**0.00**	**0.00**	0.07	**0.04**
Maó	0.04	0.05	0.01	0.02	**0.15**	**0.2**	0.05	0.02	0.01	0.02	0.01	0.00	0.00	0.01		1.00	1.00	1.00	**0.02**	**0.00**	**0.00**	**0.00**	0.05	0.30
Na Foradada	0.03	0.02	0.05	0.08	**0.12**	**0.14**	0.03	0.02	0.01	0.03	0.06	0.02	0.06	0.03	0.01		1.00	**0.02**	**0.03**	**0.00**	0.05	**0.00**	**0.04**	0.33
Pantaleu	0.01	0.01	−0.01	0.07	**0.11**	**0.14**	0.02	0.00	0.01	0.01	0.03	−0.01	0.05	0.05	0.00	0.00		1.00	1.00	**0.00**	**0.00**	**0.00**	**0.00**	**0.02**
Columbretes	**0.1**	0.1	0.01	0.01	**0.25**	**0.29**	**0.09**	0.04	0.03	0.01	0.02	0.01	0.03	0.06	0.02	0.1	0.06		**0.03**	**0.00**	**0.00**	**0.00**	0.06	**0.01**
Palomas	**0.08**	0.1	**0.11**	0.09	**0.19**	**0.22**	**0.12**	**0.14**	0.1	**0.11**	0.08	0.07	0.07	0.11	0.05	**0.09**	0.09	0.12		**0.00**	**0.00**	**0.00**	**0.00**	**0.00**
Chafarinas	0.02	0.01	0.03	0.09	**0.1**	**0.1**	0.04	0.04	0.02	0.06	0.04	0.00	0.05	0.08	0.02	0.03	0.00	0.08	0.07		0.33	**0.00**	**0.00**	**0.00**
**Atlantic**																								
Berlenga	**0.09**	0.1	**0.21**	**0.25**	**0.1**	0.08	**0.15**	**0.18**	**0.19**	**0.25**	**0.23**	**0.16**	**0.19**	**0.24**	**0.18**	**0.14**	**0.16**	**0.32**	**0.19**	**0.1**		1.00	**0.00**	0.39
Canarias	**0.09**	0.09	**0.18**	**0.23**	**0.09**	**0.08**	**0.14**	**0.17**	**0.17**	**0.24**	**0.21**	**0.14**	**0.19**	**0.21**	**0.18**	**0.15**	**0.16**	**0.28**	**0.2**	**0.1**	0.02		**0.00**	**0.00**
Selvagem	0.07	0.06	**0.06**	0.06	**0.23**	**0.26**	**0.1**	0.07	0.04	0.04	0.00	0.04	0.06	0.06	0.01	0.07	0.07	0.09	0.09	0.06	**0.2**	**0.19**		1.00
Faiol (Azores)	**0.13**	0.1	**0.11**	0.11	**0.26**	**0.29**	**0.15**	**0.1**	0.09	0.08	0.06	0.09	0.09	0.08	0.06	0.07	0.11	0.16	0.12	0.09	**0.21**	**0.21**	0.02	

F_ST_ values [47] are given below the diagonal whereas the P values for exact tests of genotypic differentiation across all loci after Benjamini- Yekuteli corrections are given above the diagonal. Significant values after permutation test (>1000 times), and after Benjamini- Yekuteli corrections (P<0.005) are shown in bold. For the location of colonies, see Fig. 1.

When looking at the genetic differentiation between the Atlantic and Mediterranean colonies, significant F_ST_ pairwise differences appeared even though some were not statistically significant after Benjamini-Yekuteli corrections (Table 2). However, compared to previous results from mtDNA (F_ST_ = 0.58) these differences were lower than expected. Differences between mtDNA and microsatellites might be a simple consequence of different coalescent time for markers ([69]; but see [70]), thus we also calibrated F_ST_ values for comparisons (F_ST_ = 0.26) [71]. Individuals from Canaries and Berlenga were the most differentiated from the Mediterranean birds (mean F_ST_ = 0.18 and 0.17, respectively), being statistically significantly distinct from almost all Mediterranean colonies (Table 2). Individuals from Azores and Selvagem were also statistically significantly distinct from many Mediterranean colonies but not from others (Table 2). In addition, Spargiotto and Barretini populations were genetically closer to those from the Canaries and Berlenga than from the other Mediterranean colonies (Table 2).

**Figure 2:**
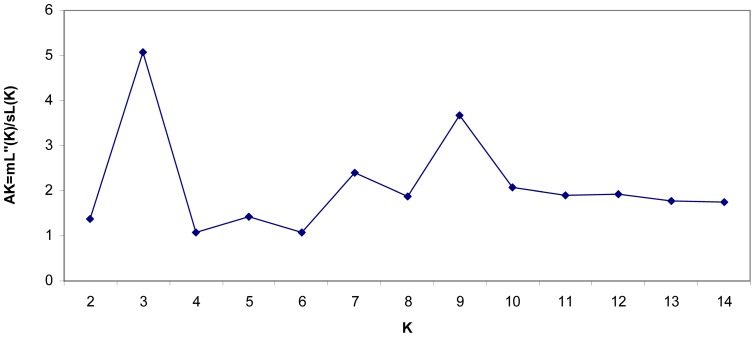
Detection of the number of groups in the data set with Structure (see [51]), with ΔK as a function of K.

**Figure 3:**
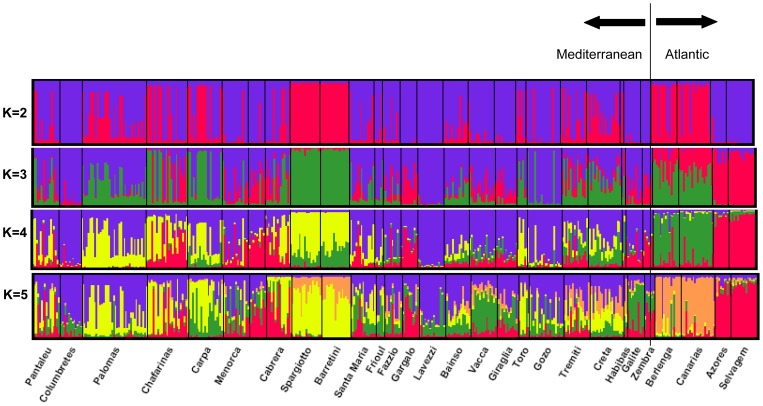
Bayesian clustering of Cory's shearwater genotypes performed in Structure with K = 2, K = 3, K = 4 and K = 5. Each individual is represented by a vertical line, with the probability of assignment to different clusters. Bold vertical lines separate breeding colonies.

Three population clusters (K = 3) were identified when applying the Bayesian clustering approach implemented in Structure (Figure 2, 3), although the relationship between populations and in particular, the assignment or membership of each cluster did not follow a simple geographical interpretation. Results for larger K values were consistent with results for K = 3 (Figure 3). While some individuals were strongly assigned to one particular population (e.g. most individuals from Barretini, Lavezzi or Selvagem), many individuals from Mediterranean populations and from Canarias and Berlenga did not (Figure 3). As in previous analyses, a clear differentiation appears between Atlantic individuals from Canarias-Berlenga and those from Selvagem-Azores.

In the AMOVA analysis, we detected a low but statistically significant global genetic structure (F_ST_ = 0.15, P<0.0001). The hierarchical AMOVA showed the highest significant F_CT_ value when the sample sites were divided into the four groups corresponding to the two Atlantic groups, Spargiotto and Barretini, and all the other Mediterranean colonies (Table 3).

**Table 3:** Hierarchical analysis of molecular variance (AMOVA).

Genetic structure	F_SC_	P value	F_CT_	P value
2 Groups: Medit; (Atl1, Atl2)	0.079	<0.001	0.046	0.003
3 Groups: Medit; Atl1; Atl2	0.069	<0.001	0.080	0.003
3 Groups: Medit -(Spa, Bar); Atl1; Atl2 + (Spa, Bar)	0.05	<0.001	0.103	<0.001
4 Groups: Medit -(Spa, Bar); (Spa, Bar); Atl1; Atl2	**0.044**	**<0.001**	**0.112**	**<0.001**
Without grouping	F_ST_ = 0.15	<0.0001		

F_CT_ is defined as the variance among groups divided by total variance, F_SC_ is the variance among populations divided by the variance among and within populations and F_ST_ is the variance among groups and among populations divided by total variance [55]. The highest significant F_CT_ is in bold. Medit: Mediterranean, Atl.1: Faiol and Selvagem, Atl.2: Canaries and Berlenga. Spa: Spargiotto, Bar: Barretini.

**Figure 4:**
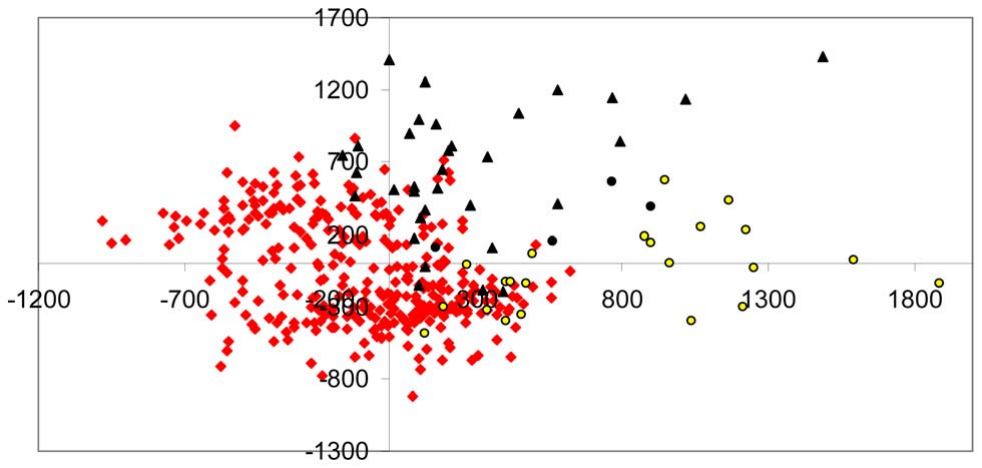
Factorial Correspondence Analysis of nuclear microsatellite variation. Plot of the first two axes (factors) of a factorial correspondence analysis (AFC) based on allelic variation at six microsatellite loci for 400 Cory's shearwaters. Triangles represent individuals from Berlenga and Canaries, circles individuals from Selvagem and Azores, and squares individuals from Mediterranean colonies.

The representation of the MCA on the two principal axes is shown in Figure 4. The first axis of the MCA allowed us to differentiate between Atlantic and Mediterranean individuals, even if a high level of admixture was detected between subspecies. As previous genetic analyses have shown (Pairwise genetic distance and AMOVA), the second axis revealed a genetic differentiation between Atlantic colonies.

### Gene flow analyses

When analysing the short-term gene flow between basins using BayesAss we found that a high proportion of individuals derived from their own population (approximately 90% and 97% in the Mediterranean and the Atlantic colonies respectively); we detected some recent gene flow between basins, about 3% from then Mediterranean to the Atlantic (m = 0.0297; 95% CI 0.001–0.100) and about 10% from the Atlantic to the Mediterranean (m = 0.0998; 95% CI 0.004–0.226), suggesting that low but effective dispersal has occurred recently between subspecies. When analysing the long term gene flow using Migrate, theta values in the Mediterranean and Atlantic basins were 10.173 (0.667–18.346) and 4.503 (0.000–9.6731), respectively. We detected some historical dispersal between basins, being M =  9 (95% CI 0.000–21.333) and M = 12.333 (95% CI 0.000–26.667) the estimated scaled-migration rates from the Atlantic to the Mediterranean and from the Mediterranean to the Atlantic, respectively. However the results should be treated with caution because 95% confidence interval estimates include zero in both cases. We estimated that each generation ∼90 individuals migrated from the Atlantic to the Mediterranean and 55 individuals migrated from the Mediterranean to the Atlantic, with a mean of 70 individuals exchanged between basins per generation.

### Spatial analysis

**Figure 5:**
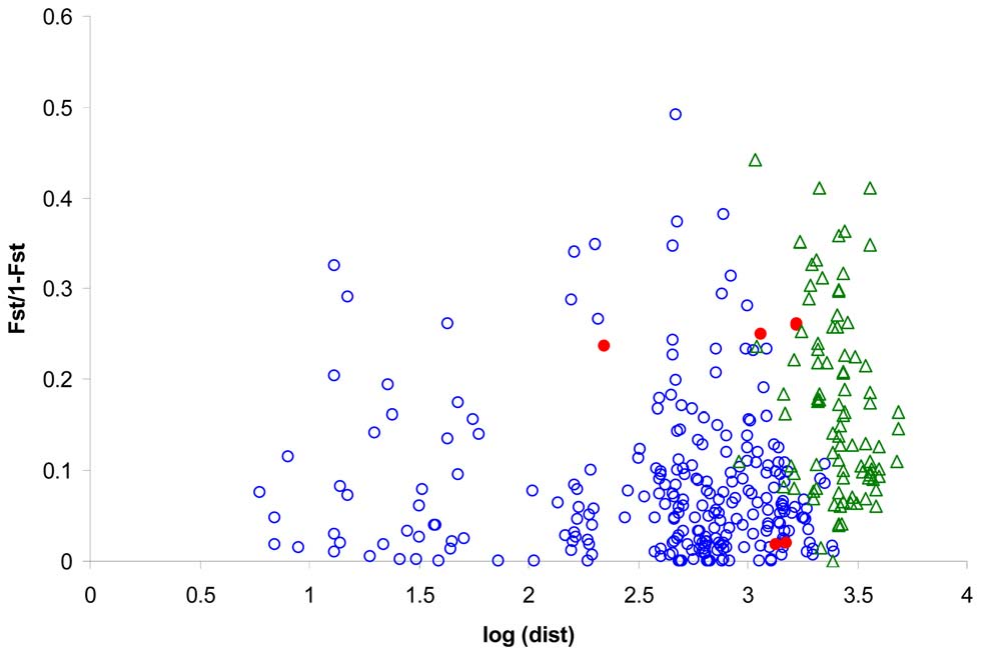
Genetic and geographic distances for pairs of sampled geographic areas. Spatial autocorrelogram for Cory's shearwater. Blue circles indicate comparisons between pairs of Mediterranean sampled colonies, red dots indicate pairs of Atlantic sampled colonies and green triangles indicated pairs of one Mediterranean sampled colony and one Atlantic colony.

When all Atlantic and Mediterranean colonies were analysed, the slope of the regression between distance matrices was estimated to 0.027, with a slightly significant association between matrices (P = 0.025) and suggesting a low isolation by distance pattern. We can see in Figure 5 that genetic differentiation in some interbasins comparisons are great, but in some others differentiation is lower than differentiation between Mediterranean colonies. However this pattern completely disappeared when only Mediterranean colonies were analysed, obtaining an estimated slope of −0.009 (P = 0.55).

## Discussion

### Genetic differentiation and dispersal within basins

We found that much greater genetic population structuring was present in the Atlantic than in the Mediterranean. The similarities between individuals from Berlenga and Canaries on one hand, and between Selvagem and Azores, on the other hand, were not evident with mtDNA analysis and are surprising if we bear in mind the geographical locations of these archipelagos. Nevertheless, previous comparisons of morphometric data from adult birds from Berlenga and Selvagem Grande did detect significant differences in all characters measured (including eggs) except for wing-length, even though both populations belong to the subspecies *borealis* [72]. The fact that genetic differences were detected in our study suggests that these morphometric differences may not simply originate as a result of different ecological conditions, but may be also due to genetic factors. We suggest that a possible cause of the close genetic similarities between Berlenga-Canaries and Selvagem-Azores populations may lie in extensive recruitment from one of these colonies after recent human persecution. Another non exclusive explanation would be that greater connectivity due, for example to different wind patterns, may have allowed birds to move easily between these colonies (see [14], [73]). Interestingly, the stronger population structure in the Atlantic subspecies was noted when analyzing DNA fingerprinting [18], and led these authors to suggest that the Mediterranean subspecies had only recently radiated from a founder group of Atlantic individuals. However, we suggest that the differences in population structure between subspecies may be also due to different patterns of dispersal. Nonetheless, if we are to fully understand the genetic differentiation and dispersal patterns within the Atlantic subspecies, genetic and ecological studies that include more Atlantic colonies should be carried out to.

### Genetic differentiation and dispersal between basins

Patterns of genetic variation in Cory's shearwater revealed differences between the Atlantic and Mediterranean colonies which were lower than expected, especially compared to previous studies of Mt DNA. Capture-recapture data in this species suggests mainly local recruitment and, less frequently, low-to-medium-range dispersal [14], [26], [74], [75]. However, occasional movements of individuals between distant colonies within and between Mediterranean and Atlantic colonies have been reported ([16], [29]–[32], author's unpublished data). Gómez–Díaz et al. [14], for example, found that 97% of the resighted birds recruit to their natal colony, 2% dispersed into neighbouring breeding sites less than 300 km away, and less than 1% dispersed distances greater than 1000 km. Interestingly, Gómez–Díaz et al. [14], found that among the long-distance movements there were 4 interbasin dispersal events: 3 birds from the Atlantic moved into the Mediterranean, and 1 bird moved from the Mediterranean into the Atlantic. Also the proportion of Atlantic individuals breeding in the Chafarinas Islands from 2000 to 2010 has increased over the years from 6% to a 23% (personal observations). Thus it seems that our genetic analysis of the gene flow between basins, would agree with capture-recapture data: most individuals would derive from their own basin, but there would be also some dispersal between basins. This dispersal pattern with rare but recurrent long-distance dispersal events has recently been proposed for other Procellariformes [8], [76] and also agrees with previous capture-recapture studies conducted on this group of seabirds [77], [78]. However, as recently suggested in Bicknell et al. [79], for such vagile species with large populations, other complementary approaches than genetic ones are needed to more confidently assess dispersal rates.

### Implications for taxonomy

Small genetic differences between subspecies should not simply be interpreted as evidence of high connectivity [80], however gene-flow analyses between basins, specially the short-term analysis, also suggest dispersal between subspecies. This raises a further question about species identity since it has recently been suggested that these two subspecies do in fact represent, two separate species (e.g. [14], [36]).

From a morphometric point of view both Atlantic and Mediterranean taxa are clearly differentiated [14]. However, morphological variation is clinal inside the Mediterranean [81], some western populations are similar in size to those of the Atlantic, and strong morphometric differentiation exists within the Atlantic taxa. Also vocalizations show differences between taxa [35]. However, it would seem that differences in vocalizations would not act as a reproductive barrier as a male with Atlantic accent mated with a Mediterranean female on a Mediterranean colony [29]. Additionally, also at a lower scale, vocalizations differ between archipelagos and islands [73], showing the existence of a geographic variation of acoustics parameters [35], [75].

Phylogenetic analyses of mitochondrial DNA suggested that both subspecies formed reciprocally monophyletic groups and they estimated the gene flow between basins to be less than 1 female per generation [14]. However they also identified 4 out of 241 birds in which the mitochondrial haplotype did not match the breeding area; they found two genetically Atlantic birds breeding in the Mediterranean with an Atlantic phenotype, suggesting two migration events, and two cases were the phenotype and genotype did not match, suggesting introgression. Additionally in the Chafarinas Islands the proportion of Atlantic individuals breeding in the colony from 2000 to 2010 has increased over the years from 6% to a 23% and more important, about 14% of the monitored couples resulted from mixed pairs (authors' unpublished data). Thus we suggest that gene flow between these two taxa could be higher than previously estimated with mitochondrial DNA. In our opinion the taxonomic debate is still open and more data is needed to conclude if these two taxa should be regarded as two different species or subspecies.

### Spatial patterns of dispersal

Only a slight IBD pattern appeared when Atlantic and Mediterranean colonies of Cory's shearwaters were analysed, suggesting that the geographical distance between breeding colonies may not be the primary determinant of population divergence. The IBD pattern completely disappeared when analyzing only Mediterranean colonies, and this may not be due to a difference on sample sizes between analyses [67], as in this case they did not vary greatly (from 27 to 24 colonies). This suggests that different processes are at work in genetic divergence at small and at large geographical scales. For example, some colonies in the central Mediterranean (Spargiotto and Barretini) had a strong genetic relationship with them but also with Atlantic birds, whilst other Mediterranean colonies, less than 1 km away, did not (see Fig 1). The role of intersexual acoustic signals in species or subspecies discrimination has been shown to exist in birds [82], [83] and even in shearwaters [84], [85], and so attraction by conspecifics [86], for example via vocal recognition, may be influencing effective dispersal in this species. Additionally, previous studies have suggested that wind plays a major role in determining migratory routes in this species [87], [88] and so prevailing winds are probably a better reflection than absolute distances between colonies of the connectivity between geographic locations. The features of natal colonies (e.g. local extinction, introduction of predators, nest availability) may also affect dispersal patterns. Therefore the genetic structure of populations may be determined not only by the species' dispersal capacity and its philopatric tendencies but also by other physical and biotic factors and environmental shifts.

Previously the Almería-Oran Oceanic Front (AOOF), rather than the Straits of Gibraltar, was proposed as the phylogenetic break between Cory's shearwater taxa [16]. The rationale of this conclusion is the presence of a Mediterranean colony (Almería) placed in front of the (AOOF), in which all individuals are genetically (mtDNA) and morphologically Atlantic. Unfortunately we were not able to include any samples from this colony in our study. Nevertheless data exist that would seem to question the role of the AOOF as the phylogeographic break between subspecies: first we detected a strong Atlantic imprint in the two colonies located in the middle of the Mediterranean (Spargiotto and Barretini) and, second, most individuals in a colony placed before the AOOF (Chafarinas), had Mediterranean characteristics (see also [16]). Thus, we suggest that more information is still needed to confirm or reject the importance of the AOOF in the population structure of this species.

## Conclusions

Our results are consistent with the limited capture-mark-recapture data and suggest that in Cory's shearwater: i) distance is not the main factor in shaping population structure, ii) local recruitment is the most frequent dispersal event, and iii) there are rare but recurrent long-distance dispersal events. We conclude that dispersal between subspecies may not be negligible, thus, in contrast to recent work, we recommend gathering more data to determine whether these taxa should be considered two different species or subspecies.

## Supporting Information

Table S1 **Amplification conditions for microsatellite primers.** Conditions were designed for microsatellite amplification in Balearic shearwater and used in Cory's shearwater; for primer sequences see González et al. (2009).
(DOC)
