# Supplementary material for: Population Structure and Dispersal Patterns within and between Atlantic and Mediterranean Populations of a Large-Range Pelagic Seabird
Source: PLoS One. 2013 Aug 12;8(8):e70711. doi: 10.1371/journal.pone.0070711 (PMC3741395; doi:10.1371/journal.pone.0070711)
Supplement: Table S1 — Amplification conditions for microsatellite primers. Conditions were designed for microsatellite amplification in Balearic shearwater and used in Cory's shearwater; for primer sequences see González et al. (2009). (DOC) [file pone.0070711.s001.doc]

|  | Primer | Size | Amplification conditions |
| --- | --- | --- | --- |
| Polymorphic |  |  |  |
|  | puff 1.9 | 131-133 | T anealling 52ºC, 2 mM Mg |
|  | puff PM2 | 172-194 | T anealling 55ºC, 3 mM Mg |
|  | puff C5A | 184-186 | T anealling 58ºC, 3 mM Mg |
|  | G2F | 200-243 | T anealling 60ºC, 2 mM Mg |
|  | G11F | 173-187 | T anealling 60ºC, 3 mM Mg |
|  | G2C | 149-169 | T anealling 50ºC, 3 mM Mg |
| Not polymorphic |  |  |  |
|  | puff D1B | 167 | T anealling 58ºC, 3 mM Mg |
|  | puff 1.5 | 169 | T anealling 53ºC, 3 mM Mg |
|  | puff C5D | 172 | T anealling 58ºC, 3 mM Mg |
